# Medical students’ use of Facebook for educational purposes

**DOI:** 10.1007/s40037-016-0273-5

**Published:** 2016-06-06

**Authors:** Anam Ali

**Affiliations:** Barts and the London School of Medicine and Dentistry, London, UK

**Keywords:** Facebook, Medical education, e-learning, Undergraduate

## Abstract

Medical students use Facebook to interact with one another both socially and educationally. This study investigates how medical students in a UK medical school use Facebook to support their learning. In particular, it identifies the nature of their educational activities, and details their experiences of using an educational Facebook group. Twenty-four medical students who self-identified as being Facebook users were invited to focus groups to attain a general overview of Facebook use within an educational context. A textual analysis was then conducted on a small group of intercalating medical students who used a self-created Facebook group to supplement their learning. Five of these students participated in semi-structured interviews. Six common themes were generated. These included ‘collaborative learning’, ‘strategic uses for the preparation for assessment’, ‘sharing experiences and providing support’, ‘creating and maintaining connections’, ‘personal planning and practical organization’ and ‘sharing and evaluating educational resources’. Evidence from this study shows that medical students are using Facebook informally to enhance their learning and undergraduate lives. Facebook has enabled students to create a supportive learning community amongst their peers. Medical educators wishing to capitalize on Facebook, as a platform for formal educational initiatives, should remain cautious of intruding on this peer online learning community.

## Essentials

- Facebook is playing a significant part in contemporary medical education; however, its use as a formal platform or an informal adjunct is debatable.
- Medical educators wishing to capitalize any kinds of educational initiatives on Facebook, should remain sensitive of intruding on this social online learning community, so natural student dynamics continue to function.

## Introduction

Prensky [1] defines ‘digital natives’ as a generation of people who have grown up with online digital technologies and use them frequently in their everyday lives. This includes the use of online social networking sites, which have become prominent platforms of the 21st century.

Facebook is an example of an online social networking site which allows friends and families to share information and stay connected with one another [2]. Since its launch, Facebook has increased in growth, and has over one billion monthly active users worldwide as of June 2015 [3]. Whilst the underlying concept is not that of a learning environment, Facebook’s tools and features can serve as a valuable support to academic activities [4]. Built-in features allow interaction via ‘profiles’, ‘groups’ and ‘pages’ and communication with individuals or groups using online chat, video chat and inbox messaging. User profiles are personal spaces through which personal information, photographs and videos can be shared with an online community. Groups are dedicated spaces for small group communication about shared interests. They can be created by anyone and have variable privacy settings. Pages are visible to the public and allow official entities such as public figures, businesses and organizations to communicate broadly with members who like them [5].

In recent years, there has been significant interest amongst medical educators about the educational uses of Facebook. A survey conducted by Sandars et al. [6] identified that 70 % (*n* = 212) of medical students used social networking sites including Facebook. In a newer study, Bosslet et al. [7] purports this figure to be 90 % (*n* = 1004). Gray et al. [4] devised a mixed-methods study, which reported that 25 % (*n* = 660) of medical students used Facebook to support their learning. Activities included facilitating exam revision, sharing resources, supporting tutorial group learning and keeping in touch throughout studies. Furthermore, 50 % of those who did not currently use Facebook educationally were open to the idea of using it. The authors suggest that perhaps the study may be limited to some degree, as the educational-related uses were likely to be under-reported. In another paper, Selwyn [8] observed and analyzed Facebook wall postings of social science undergraduates. Only 4 % (*n* = 68,169) of the total content pertained to academic-related activity within which five main themes emerged: ‘exchange of academic information’, ‘exchange of practical information’, ’reflections on the university experience’, ‘displays of supplication’ and ‘banter’ (humorous exchanges). He conceded that staff should abstain from intervening in such social environments to allow them to continue ‘backstage’.

The use of Facebook groups and pages has also been explored. Estus [9] conducted an evaluation on the use of a closed Facebook discussion group within a pharmacotherapy course. Over half of the students (*n* = 28) agreed that the Facebook activities were valuable and supplemented the course. Recently, Jaffar [10] described the use of a Facebook page to enhance human anatomy education. Survey analysis revealed that 89 % (*n* = 118) of the students perceived the page to be effective in contributing to their learning experience.

Some authors have suggested additional uses of Facebook including encouraging medical students to engage in reflective practice, teaching professionalism [11], usage as a stress management support system [12], and cultivating interest in research [13]. Furthermore, a number of authors discuss how the online environment of Facebook has the potential to facilitate collaborative and peer learning [14, 15], and the achievement of clinical excellence [16] within medical education.

This purpose of this study is to identify and detail the specific kinds of educational activities that medical students partake in through Facebook using an in-depth qualitative design. Moreover, it seeks to explore the use of a student-created informal Facebook group as a vehicle for enhancing learning in a medical education course. It is hoped that this will strengthen and complement existing literature, and broaden medical educators’ understanding of the role that Facebook plays in contemporary medical education.

## Method

### Study setting and design

This study was conducted at Barts and the London School of Medicine and Dentistry during the 2011–2012 academic year. The university follows a five-year MBBS medical programme. After Years 2–4, students can undertake an optional one-year intercalated degree leading to an additional BSc qualification.

A qualitative approach was favoured, in order to generate data that reflected the in-depth views of the participants. Focus groups (Phase 1) were followed by semi-structured interviews (Phase 2) to allow triangulation of data (Table 1; [17]). Ethical approval was obtained from the Queen Mary University Ethics Committee. Participants were volunteers recruited through email, Facebook and lecture announcements.

**Table 1:** Study design

Phase 1	*Focus groups* General overview of medical students use of Facebook for educational purposes Participants: Medical students from the undergraduate medical programme, Years 1–4
Phase 2	*Semi-structured interviews* Focused case study on how a student-created educational Facebook group was used by a small group of medical students Participants: Medical students from the intercalated degree in Medical Education

### Phase 1

Focus groups were conducted to attain a general overview of how medical students use Facebook for educational purposes. A purposive sample of undergraduates from the medical programme who self-identified as being Facebook users were selected. Students were sampled from all year groups to collate a broad selection of views and experiences. It was, however, difficult to recruit Year 5 students due to upcoming exams. Four focus groups were conducted, lasting 40 minutes on average. A total of 24 medical students (female, *n* = 10; male, *n* = 14) were recruited. The number of participants ranged from four to seven per focus group (Year 1, *n* = 6; Year 2, *n* = 4; Year 3, *n* = 7; Year 4, *n* = 7).

### Phase 2

To explore the subject in greater depth, a focused textual analysis was conducted on a small group of undergraduates (*n* = 12) from the intercalated degree in medical education, who used an educational Facebook group to supplement their course. The group was created by a student on the course before the start of the academic year in September 2011 (Table 2). The primary researcher was a student on the course and therefore a group member with access to the group. Five intercalating students (female, *n* = 3; male, *n* = 2) volunteered for semi-structured interviews, which lasted 20 minutes on average. Online postings of the Facebook group were also analyzed. On inception of this study in March 2012, written consent was obtained from all members for permission to analyze the group, and only preceding posts were reviewed. Prior to this students were not aware their posts would be examined. All posts of the primary researcher were excluded from the analysis.

**Table 2:** Characteristics of intercalating students’ online Facebook group

Aim	‘Just thought it would be good to make a group so we can all get to know each other, and it may come in handy next year if we need to contact one another about work/projects etc.’
Context	Informal student-created group for students (*n* = 12) taking the intercalated degree in medical education ‘Secret’ group: found in the search bar only by members Membership by invitation only No faculty present Controlled by the students
Rules	No formal rules established
Tools and features	Discussion ‘wall’ allowing users to post comments, all commentary activity occurred here Create events and create polls Share files and documents Upload photos and videos

### Data collection and analysis

The themes and gaps identified from the literature review informed the design of a topic guide. Openly framed questions were used to structure the discussions [17]. Participants were given information sheets, written consent was obtained prior to data collection and anonymity was guaranteed. Audio recordings of the focus groups and interviews were transcribed verbatim. The transcripts were checked against the recordings for accuracy.

Data collection and analysis was carried out between March and April 2012 by the primary researcher. The two sets of data were analyzed separately, using thematic analysis to explore in-depth personal perspectives of the students through text interpretation and the systematic classification of patterns [18–20]. Initially, the data were read and reread to enhance familiarity with the content. Using open coding, primary categories were identified. Connections between the categories were further explored until the main themes emerged. The data analysis process was repeated twice to reduce error and researcher bias, prevent data from being missed, and allow clarity of ambiguous data. Following this, analysis of the Facebook group was carried out. Screenshots of each online post on the main discussion wall were reviewed and categorized into the final generated themes.

## Results

Although the focus groups and semi-structured interviews were analyzed separately, both sets of data generated six common overlapping themes. Each theme is discussed and illustrated with relevant quotes, which were taken verbatim from the transcripts. Screenshots from the online Facebook group have been provided for the case study participants, analyzed in Phase 2 of the study.

### Collaborative learning

**Fig. 1:**
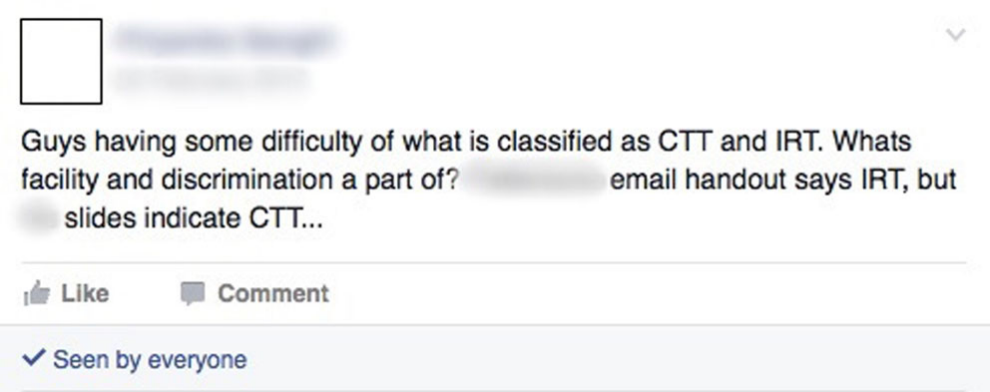
Screenshot example of collaborative learning

The transcripts included evidence of collaborative learning occurring on Facebook (Fig. 1). Students used their profiles, groups, and pages to post questions and discuss work.

“… There’s a discussion going where someone throws up a question as their status. I’ve done it before … if you don’t understand something in a lecture … any question that you’re doing or anything that you’re studying, you don’t get it … Just throw it up as a status and there’s bound to be someone up there who will comment on that status and explain it to you fully …” (MBBS focus group 2).

Topics were of an educational nature in which students asked peers for help, shared ideas and gave feedback. The resulting commentary motivated students to research the related topic and generated discussions containing humour, disputes and explanations. Through this, there was evidence of peer teaching and a shared learning experience occurring on Facebook.

“… Someone will be like, what is this? And then there’ll be long descriptions of what it is … before the exams and before the last couple of deadlines, we did a three-way message …oh, I don’t understand this, explain it… yeah, it’s like a constant conversation …Then a long explanation and I found that getting it explained by someone else really helps as opposed to the course materials, because sometimes the course materials are not as clear and it is better to hear it in your own language …” (Case study participant 4).

### Strategic uses for the preparation for assessment

**Fig. 2:**
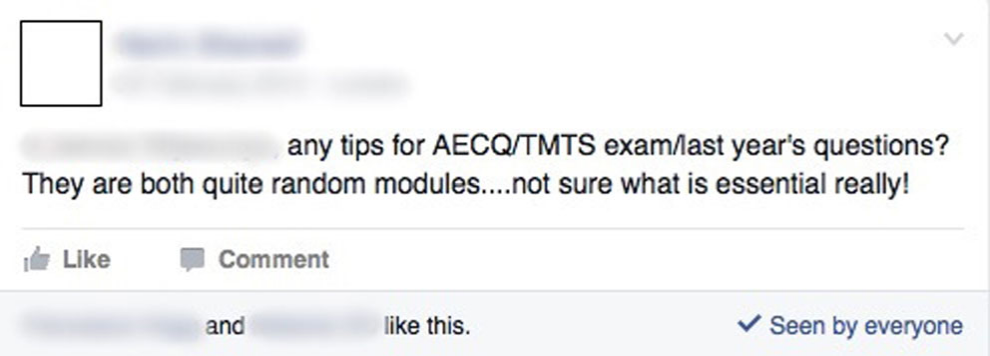
Screenshot example of strategic uses for the preparation for assessment

The data revealed a form of strategic learning happening on Facebook, which was used to exchange examples of exam questions, answers and revision material to prepare for assessments (Fig. 2). Students discussed the existence of groups and pages, some of which were student created and some by external organizations.

“ … I had a friend post on my wall … do you know how to calculate pain intensity ratios for the BNB exam? And I hadn’t even thought to revise it. So I was like, oh, I’ve never heard of that. And I went and got my handbook for the session. Took it out, copied it out verbatim and said that’s how to calculate it. And I got my head around it. And other people commented and people started liking it and people got involved in the discussion. And it actually came up as one of our PBL SAQ’s in the exam … ” (MBBS focus group 2).

“ … Also there’s the question bank … people just post … ideas of what questions they’d seen and what modules you’re going to get …” (MBBS focus group 2).

### Sharing experiences and providing support

**Fig. 3:**
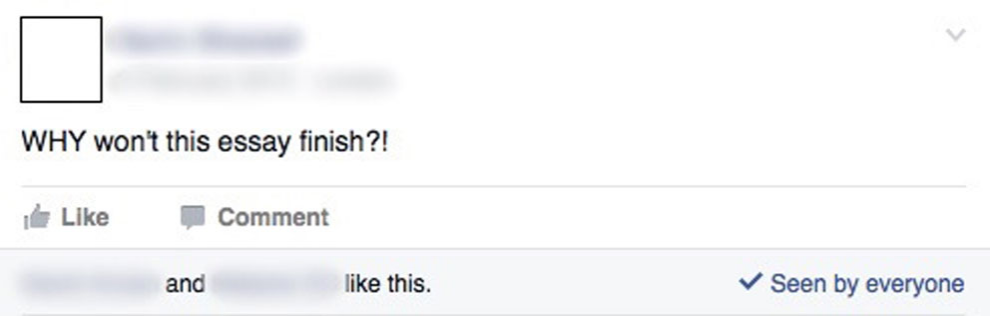
Screenshot example of sharing experiences and providing support

Students used Facebook as a platform to share feelings with one another about course experiences (Fig. 3). They motivated each other before exams, expressed emotions including frustration, dissatisfaction, and excitement and provided support to one another.

“… You’re all going through this together. You see other people’s status updates and what other people are talking about and you share experiences that you’ve had. You feel that you’re all in it together and you’re not alone, that there are other people facing the same difficulties as you, maybe studying for exams, being ignored in firms by consultants and things like that … failing to take bloods …” (MBBS focus group 3).

Assignments, assessment and administration of the course were some of the reasons, which generated the positive, negative and supportive commentary. Participants in the case study intimated that, because of the “secret” nature of the Facebook group, and small group size this theme was heavily recurring.

“… People are in the same position … and when you’re doing assignments you’ve got someone there to talk to about it, so I think without I probably might not have done as well on maybe my assignments and I might not have had the motivation to revise as much or I wouldn’t have had things cleared up for me … as easily, so I might not have sorted those out as easily as I could have done through this group …” (Case study participant 5).

### Creating and maintaining connections

The dataset showed that Facebook assisted in creating, maintaining and strengthening peer connections over the course of university life. It was a useful medium to keep up-to-date with extracurricular activities, social events and societies.

“… The group is kind of a way to stay in touch with people, know what they’re doing and know how other people are progressing with the coursework and stuff. We’re not seeing each other every day … during holidays, during study leave, before our exams, because not everyone is on campus so yeah, I think it’s really helpful ….” (Case study participant 4).

Developing peer connection was particularly helpful for the Year 1 and external students joining a new large cohort. With the case study participants, the Facebook group helped enhance their social bonds.

“… Especially as an external student I didn’t really know anyone and I wouldn’t have felt comfortable kind of texting people for information like all the time … It’s not so much of a problem now, but like at the beginning when I didn’t really know people I wasn’t really comfortable with doing that, so it’s really useful in letting me get to know everyone …” (Case study participant 4).

### Personal planning and practical organization

**Fig. 4:**
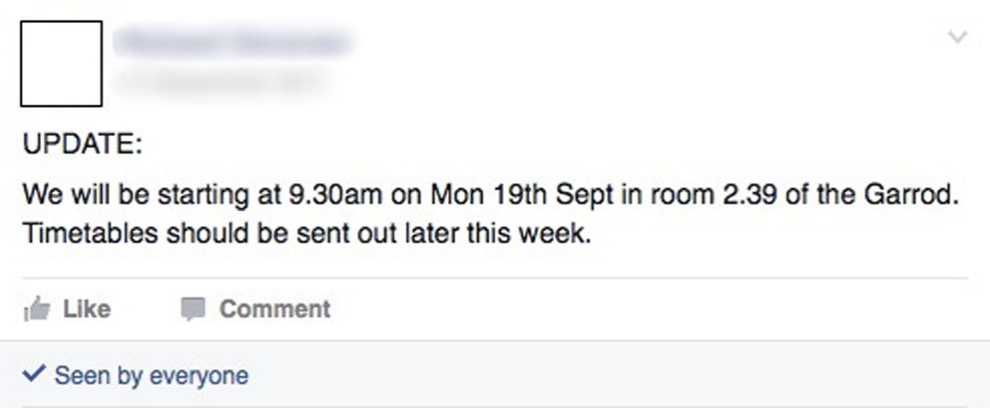
Screenshot example of personal planning and practical organization

A frequently occurring theme in the case study was the use of Facebook to exchange practical information surrounding undergraduate organization (Fig. 4). Most commonly this took the form of circulating and discussing timetable updates, attendance issues, assessment details, course announcements, staff emails and group work scheduling.

“… We found it quite good for organizing dates and things … it’s quite good just to have a forum where you can write it all down … just to keep everyone up to speed on how we’re going along. And then when the deadline got closer, organizing things like make sure we’re there at whatever time and stuff like that …” (MBBS focus group 1).

The case study students found the sharing of information on Facebook particularly beneficial, as the posting of one message communicated to the whole group instantaneously. Moreover, most students had the Facebook application on their smartphone and would get notifications immediately.

“… Without the Facebook group we wouldn’t be sure about deadlines and stuff because we help each other … with the work as well, so I think it would be a huge loss without it …” (Case study participant 3).

### Sharing and evaluating educational resources

**Fig. 5:**
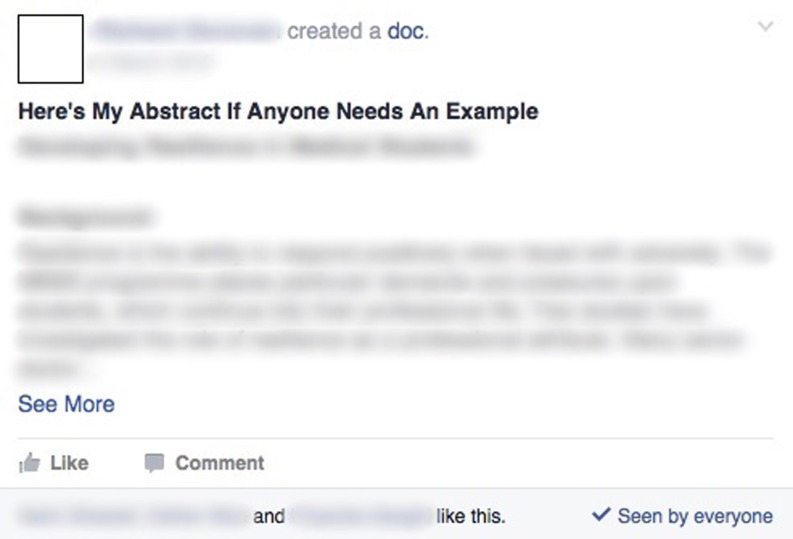
Screenshot example of sharing and evaluating educational resources

It is important to re-acknowledge that the students in this study are ‘digital natives’, who prefer online resources as their primary source of information. This theme highlights the transmission and sharing of academic resources by students through Facebook (Table 3; Fig. 5). Supporting literature recognizes that although most medical content has remained the same from year to year, the ways in which it is presented, organized and disseminated has evolved considerably. Sharing material has progressed from handwritten notes and textbooks to a plethora of various media types including online documents, videos, PowerPoint presentations, discussion forums, and websites [21].

**Table 3:** Examples of resources shared on Facebook

Student work
Tutorial learning objectives e.g. PBL session
Lecture presentations
Educational images, websites and YouTube videos
Literary articles
Online books

## Discussion

The data generated from this study reinforce many of the findings in the literature review and provide further evidence that medical students in the UK are using Facebook for educational purposes. The key findings are that Facebook is being used for collaborative learning, assessment preparation, creating peer connections, providing support, organizing studies and sharing educational resources.

Facebook’s interface facilitates the exchange of user generated content and this creates opportunities for collaborative learning, allowing students to be participative, interactive, and social with one another [22]. Bullock [23] discusses how these processes can support learning from participation and the development of contextual knowledge. Indeed, as students share, discuss and reflect on common interests, ideas and explicit work knowledge, an online community of practice is formed [24].

It is interesting to observe the extrinsic behaviours of medical students on Facebook. The medical school in this study has a policy of prohibiting the distribution of exam questions, so it is fascinating to find that Facebook has become a trading market source for these types of materials. This highlights issues regarding the circulation of incorrect information and may have wider implications on professionalism; however, the students did not attach a negative connotation to this practice. Indeed, strategic learning is common amongst medical students due to emphasis on summative knowledge-based exams [25].

Evidence from the current study illustrates how Facebook is acting as an unofficial support network. An online profile not only makes a student more accessible and relatable, but also serves as a starting point for students to discuss similar experiences. Support networks are important in helping students to transition and adjust into medical school life, and strengthen social and academic ties [26]. Additionally, students now have the technology to remain logged onto Facebook 24 hours a day, allowing all types of student-student communication on Facebook to become instantaneous and widespread [21].

From discussions with the case study participants, and analysis of the Facebook group page, it was clear that this online forum positively complemented the intercalated degree and was a valuable resource. Although the level of activity was not quantitatively assessed, student interaction was ongoing throughout the year, with all members contributing. Student posts included intertwined educational and social commentary. The group appeared to enhance student bonding and complement their formal education. Students intimated how staff absence, optional participation and the secret informal nature of the group were important factors in effective functioning of the group, as students were able to post freely and comfortably without the scrutiny of staff. These observations correspond with Cain and Polistrici’s [27] findings, who describe the use of a Facebook group as an informal learning environment for pharmacy management students. Moreover, medical educators might be wise to consider the views of Selwyn [8], who suggested that staff should refrain from ‘educationally appropriating’ such social areas and allow them to continue ‘unabated’. He stated that, ‘allowing students the freedom to construct a set of disruptive, challenging and disengaged social identities, roles and personal biographies of doing university in an offline, backstage space such as Facebook could be seen as a vital contribution to the successful provision of offline university education’. Indeed, this study presents evidence of a hidden curriculum occurring on Facebook, in which the norms, values and beliefs of the medical school experience are shared on Facebook and the strength of its use amongst students lies in its informality, and the way it remains ‘hidden’ from the formal course organizers [28].

Although the data have generated pertinent conclusions, there are several limitations to this study. A small sample of medical students from one medical institution was used, representing a narrow spectrum of the overall study body. Those who volunteered to participate in the study may have thus held stronger views on the topic compared with the overall population. Furthermore, although reminded of anonymity and confidentiality, participants may have held concerns regarding the disclosure of academically inappropriate activity, being labelled as ‘bookworks’ for using a social platform for educational purposes by fellow medical students [4]. The primary researcher was a student on the intercalated degree and carried out the analysis. To minimize participant and researcher bias, the primary author was excluded as a participant from the semi-structured interviews, their online data on the Facebook group were excluded and the analysis process was repeated twice. Finally, the findings of this study rely on highly interpretive and subjective perspective of analysis of the data presented.

## Conclusion

The findings of this study provide medical educators with insight into their students’ online behaviours, and some foundation knowledge to inform any initiatives that use Facebook for educational purposes. It is suggested that similar studies in other institutions are conducted on a larger scale in order to assess the generalizability of the findings about medical students educational use of Facebook.

Facebook is by no means an alternative to traditional teaching methods. Indeed it must be remembered that it is a social networking site rather than a virtual learning environment. However, the findings here suggest that, in some circumstances, Facebook can be used as a complementary educational platform that allows each learner to create a personalized online learning space amidst peers. It is suggested that educators should encourage the use of secret or closed informal Facebook groups in educational contexts especially within small groups to enhance learning. This study has demonstrated evidence of students creating an informal social learning community on Facebook, detached from the formal curriculum. With staff presence and formalization, this community, along with its natural student dynamics, may cease to function. Medical educators should be sensitive to this online learning space if they are to take advantage of Facebook and engage the minds of the ‘digital natives’.

## References

[1] Prensky M (2001). Digital natives, digital immigrants part 1. Horizon.

[2] Trumble S (2010). Making sense of global issues. Clin Teach.

[3] Facebook newsroom 2015. http://newsroom.fb.com/company-info/. Accessed 18 December 2015.

[4] Gray K, Annabell L, Kennedy G (2010). Medical students’ use of Facebook to support learning: insights from four case studies. Med Teach.

[5] Facebook help centre. Desktop Help 2015. https://www.facebook.com/help/. Accessed 18 December 2015.

[6] Sandars J, Homer M, Pell G, Croker T (2008). Web 2.0 and social software: the medical student way of e‑learning. Med Teach.

[7] Bosslet GT, Torke AM, Hickman SE, Terry CL, Helft R (2011). The patient-doctor relationship and online social networks: results of a national survey. J Gen Intern Med.

[8] Selwyn N (2009). Faceworking: exploring students’ education related use of Facebook. Learn Media Technol.

[9] Estus EL (2010). Using Facebook within a geriatric pharmacotherapy course. Am J Pharm Educ.

[10] Jaffar AA (2014). Exploring the use of a Facebook page in anatomy education. Anat Sci Educ.

[11] Brown AD (2010). Social media: a new frontier in reflective practice. Med Educ.

[12] George DR, Dellasega C, Whitehead M (2012). Facebook stress management group for Year 1 medical students. Med Educ.

[13] Al-Khateeb AA, Abdurabu HY (2014). Using social media to facilitate medical students’ interest in research. Med Educ Online.

[14] Morris C, McKimm J (2009). Becoming a digital tourist: a guide for clinical teachers. Clin Teach.

[15] Ravindran R, Kashyap M, Lilis L, Vivekanantham S, Phoenix G (2014). Evaluation of an online medical teaching forum. Clin Teach.

[16] Batt-Rawden S, Flickinger T, Weiner J, Cheston C, Chisolm M (2014). The role of social media in clinical excellence. Clin Teach.

[17] Cohen L, Manion L, Morrison L (2011). Research methods in education.

[18] Burns RB (2000). Introduction to research methods.

[19] Greene J, Thorogood N (2009). Qualitative methods for health research.

[20] Braun V, Clarke V (2006). Using thematic analysis in psychology. Qual Res Psychol.

[21] Roberts DH, Newman LR, Schwartzstein RM (2012). Twelve tips for facilitating Millennials’ learning. Med Teach.

[22] Mcgee JB, Begg M (2008). What medical educators need to know about Web 2.0. Med Teach.

[23] Bullock A (2014). Does technology help doctors to access, use and share knowledge. Med Educ.

[24] Lave J, Wenger E (1991). Situated learning: Legitimate peripheral participation.

[25] Ferguson E, James D, Madeley L (2002). Factors associated with success in medical school: systematic review of the literature. BMJ.

[26] Cain J (2008). Online social networking issues within academia and pharmacy education. Am J Pharm Educ.

[27] Cain J, Policastri A (2011). Using Facebook as an informal learning environment. Am J Pharm Educ.

[28] Hafferty F, Franks R (1994). The hidden curriculum, ethics teaching and the structure of medical education. Acad Med.

